# Electrochemical and thermodynamic study on the corrosion performance of API X120 steel in 3.5% NaCl solution

**DOI:** 10.1038/s41598-020-61139-3

**Published:** 2020-03-09

**Authors:** Khuram Shahzad, Mostafa H. Sliem, R. A. Shakoor, A. Bahgat Radwan, Ramazan Kahraman, Malik Adeel Umer, Umair Manzoor, Aboubakr M. Abdullah

**Affiliations:** 1 Department of Materials Engineering, School of Chemical and Materials Engineering, National University of Science and Technology (NUST), Islamabad, Pakistan; 2 Center for Advanced Materials (CAM), Qatar University, 2713 Doha, Qatar; 3 Department of Chemical Engineering, College of Engineering, Qatar University, 2713 Doha, Qatar

**Keywords:** Corrosion, Pollution remediation

## Abstract

The present work studied the effect of temperature on the corrosion behavior of API X120 steel in a saline solution saturated with CO_2_ in absence and presence of polyethyleneimine (PEI) as an environmentally safe green inhibitor. The effect of PEI on the corrosion behavior of API X120 steel was investigated using destructive and non-destructive electrochemical techniques. The overall results revealed that PEI significantly decreases the corrosion rate of API X120 steel with inhibition efficiency of 94% at a concentration of 100 μmol L^−1^. The adsorption isotherm, activation energy and the thermodynamic parameters were deduced from the electrochemical results. It is revealed that the adsorption of PEI on API X120 steel surface follows Langmuir adsorption isotherm adopting a Physi-chemisorption mechanism. Finally, the samples were characterized using scanning electron microscopy (SEM) and atomic force microscopy (AFM) techniques to elucidate the effect of aggressiveness of corrosive media on the surface morphology and the corrosion performance of API X120 steel. The surface topography result indicates that the API X120 steel interface in PEI presence is smoother than CO_2_ with Cl^−^ ions or Cl^−^ ions only. This is attributed to the compact protective film limits the aggressive ions transfer towards the metallic surface and reduces the corrosion rate. Moreover, PEI inhibition mechanism is based on its CO_2_ capturing ability and the PEI adsorption on the steel surface beside the siderite layer which give the PEI molecules the ability to reduce the scale formation and increase the corrosion protection due to capturing the CO_2_ from the brine solution.

## Introduction

High strength low alloy (HSLA) steels are widely finding its applications in oil & gas industry, thermal power plants and in aerospace industry etc. due to its improved mechanical properties, superior corrosion resistance and weldability when compared with plain carbon steel^1–4^. Series of comparative studies have been focused on HSLA pipelines steels in order to understand the effect of alloying elements, heat treatments and other changeable factors on their mechanical properties and microstructure^5–8^. it has been reported that the yield strength of the API X120 steel is in range 780–951 MPa based on the shape of the specimen. Moreover, the tensile strength is varied from 940 to 1023 MPa and the strength is more than X120 ksi and reaches to X120 grade according to the API standards^9–11^. Corrosion is the major problem that compact the API X120 steel pipelines in the transportation of oil, gas.

There are a myriad number of studies that investigate the influence of carbon dioxide with saline solution on the corrosion mechanism of different grades of steel pipelines employed in the pipeline applications^12,13^. Additionally, it has been reported that the presence of carbonate ions in oilfield brine solution increases the corrosion rate of the pipelines^14–16^. Several parameters have been noted for the corrosion of steel in CO_2_ environment such as PH, temperature, CO_2_ pressure, flow rate and the formative scale which is mainly composed of iron carbonate. The nature of the formative scale layer and its protectiveness is diversifiable^17,18^. In the absence of the protective scale layer, the corrosion rate increases with increasing the CO_2_ concentration and temperature. However, the protective scale formation enhances with increasing concentration of CO_2_ which reduces the corrosion rate. Moreover, the solubility of the iron carbonate layer decreases with increasing the pH^19–21^. In addition, the medium under the scale layer can cause under deposit corrosion^22,23^ and that is why removal of scale or changing its morphology is recommended for preventing corrosion. The mechanical degreasing and inhibitors are the commonly applied to prevent scale formation and the other deposits like sands and sulfides in the pipelines^24,25^. Corrosion inhibitors are frequently added to the corrosive media in order to reduce the aggressive attack on the materials and to improve their service performance^26,27^. Corrosion inhibitors are usually organic heterocyclic compounds which contains nitrogen (N), Sulphur (S), and oxygen (O) atoms having lone pair of electrons that can adsorb on the metallic surface. The adsorption of electrons blocks the metal active sites or can create a physical barrier to decrease the attachment between the aggressive ions and the metal surface^28–30^. Finsgar *et al*. investigated the effect of different molecular sizes of polyethyleneimine on the corrosion behavior of stainless-steel alloys in 3% NaCl solution. The results indicate that PEI acts as an effective corrosion inhibitor for pitting and uniform corrosion. Additionally, PEI molecule could be adsorbed in the metal surface to form a dense protective layer which acts as a diffusion barrier from different ionic species with preventing the chlorides attack from saline solution^31,32^. Meanwhile researchers reported that the PEI inhibitor with high molar mass (60,000 g mol^−1^) was highly effective in corrosion prevention of 304 stainless steel than the low molar mass (2000 g mol^−1^)^33,34^. Jianguo *et al*. confirmed that adding 50 μmol L^−1^ from PEI (50,000 g mol^−1^) gave the highest inhibition efficiency for the low carbon steel immersed in phosphoric acid^35^. Moreover, Sekine *et al*. reported that PEI molecules were not effective on reducing the corrosion rate for mild steel in cooling water that contains Ca^+^ and Cl^−^ ions^36^. Zhang *et al*. have utilized a quaternary polyethyleneimine (QPEI) as a cationic polyelectrolyte inhibitor for Q235 carbon steel in 0.5 M H_2_SO_4_. The results indicated that QPEI formed a protective layer on the metal surface which reduced the corrosion rate of iron in acidic environment. The XPS and SEM results showed that the QPEI can form a protective polymer layer on the metal surface by adsorption^37^. Additionally. Gao *et al*. differentiate between the adsorbed layer and the polymeric layer which formed from QPEI inhibitor. He hypothesized that the two layer cooperate to form a compact barrier layer on A_3_ steel surface in acidic media which prevent metal dissolution and retard H^+^ discharging^38^. Generally, polymeric compounds exhibited a noticeable corrosion inhibition merits compared to their monomer counterparts due to increasing number of active sites. However, the molecular size and steric effect could also influence the adsorption mechanism^39^.

The aim of this research work is to study the effect of polyethyleneimine (PEI) as an environmentally safe corrosion inhibitor on the corrosion behavior of API X120 in 3.5 wt% NaCl solution saturated with CO_2_ at ambient and elevated temperatures. To the best of our knowledge, this work has not been previously reported in the literature. Moreover, the adsorption isotherm, activation energy and other thermodynamic parameters were calculated from the electrochemical results. Finally, Bare (as received polished) and rusted samples were characterized by using scanning electron microscopy (SEM) and atomic force microscopy (AFM) techniques to elucidate the effect of the aggressive media on the surface morphology and thus the corrosion performance of API X120 steel. The corrosion inhibition mechanism of the PEI corrosion inhibitor on the metal interface would be elucidated based on all the obtained results.

## Experimental Work

### Materials

**Figure 1:**
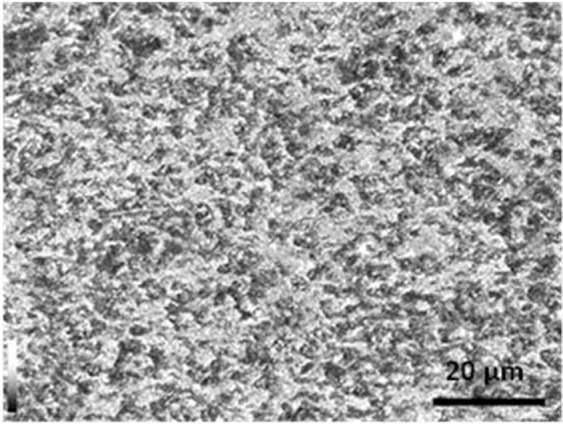
Microstructure morphology of API X120.

API X120 Steel was supplied by Shandong Yineng International Trade Company, China. The chemical composition of the studied steel was determined using an optical emission spectrometer (ARL 3460). The analyzed chemical composition is shown in Table 1. The API X120 steel was manufactured in furnace and casted it into sheets then accelerated quenching was applied reaching to the ambient temperature. The microstructure of API X120 steel sample was obtained using Lecia optical microscope, Spain. The as-received sample was etched using a freshly prepared 2% Nital solution which applied on the surface for 20 sec. Figure 1 shows the representative microstructure of the target specimen as it was found to be pearlitic. On the other hand, the mechanical properties for the API X120 steel is going to be studied in other studies.

**Table 1:** The chemical composition for API X120 steel.

Element	C	Si	Mn	Ni	Cr	Mo	Cu	V	Fe
Weight %	0.129	0.101	0.541	0.017	0.039	0.0013	0.015	0.025	Balance

**Figure 2:**
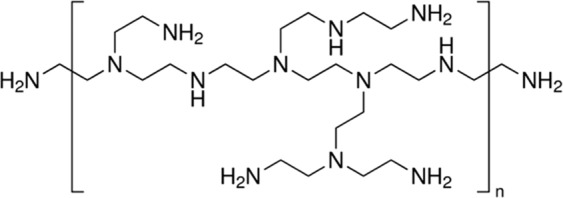
The chemical structure for the used corrosion inhibitor PEI.

Polyethyleneimine (PEI) was purchased from BDH (Germany, Project Code A11033). The chemical structure of PEI is shown in Fig. 2. The sodium chloride (NaCl) was purchased from Sigma Aldrich (Germany).

### Sample preparation and test solution

The steel specimens of size 0.5 cm^2^ were cut and grind using different size of silicon carbide (SiC) abrasive papers 500, 800, 1000 and 2000 consecutively. Finally, the ground samples were polished using 4000 grit SiC paper. Further cleaning of the polished samples was performed by acetone followed by ethanol. Finally, the samples were washed with distilled water and dried in air. The 3.5% NaCl aqueous solution was prepared by dissolving stoichiometric amount of NaCl in distilled water. The concentration of the PEI in 3.5 wt% NaCl solution was used from 25, 50, 75 and 100 µmol L^−1^. The 3.5 wt% NaCl solution was saturated with CO_2_ by purging CO_2_ into the solution at a constant flow rate. The CO_2_ purging was started 1 hour before the commencement of the experiment and remained continued though out the experiment at a constant flow rate.

### Gravimetric measurements

The weight loss experiments were performed on a API X120 steel sheet (2.0 × 2.0 × 0.1 cm) immersed in 3.5 wt% NaCl solution saturated with CO_2_ in the absence and the presence of (25, 50, 75 and 100 μmol L^−1^) of PEI corrosion inhibitor for different exposure time from 1 to 6 days at 25 °C. The tested sample were removed after the exposure time and treated according to ASTM G1-90 standards^40^.

### Electrochemical measurements

The electrochemical measurements were performed in a double jacketed cell using the three-electrode system. API X120 steel with 0.5 cm^−2^ exposed area was used as a working electrode. A graphite rod was used as auxiliary electrode and the silver/silver chloride was used as a reference electrode. The reference electrode was inserted in a lugging capillary to minimize the IR drop.

All electrochemical experiments were performed in 3.5 wt% NaCl solution saturated solution with CO_2_ containing different concentrations of PEI (25, 50, 75 and 100 µmol L^−1^) at various temperatures.at different temperatures (20, 30, 50 and 70 °C) using GAMRY potentiostat/Galvanostat (model 3000, USA). The API X120 steel was dipped into the 3.5% NaCl solution for 30 minutes to achieve the steady state for the metal with the solution before each experiment. The EIS analyses are performed under an open circuit potential (OCP) condition in a frequency range from 0.1 Hz to 100 kHz with an AC amplitude of 10 mV using a GAMRY 3000 potentiostat/galvanostat/ZRA. The potentiodynamic anodic and cathodic polarization plots were attained from −250 mV versus open circuit potential to +250 mV versus reference electrode potential at a scan rate 0.167 mV S^−1^.

### Surface characterization

The surface topography of the API X120 steel before and after corrosion measurements was examined with a field emission scanning electron microscope (FE-SEM, FE-SEM-Nova Nano-450, Netherland). In order to have more details about surface characteristics of the corroded samples, an atomic force microscope, AFM (AFM, Asylum Research, USA) was used.

## Results and Discussions

### Weight loss measurements

**Figure 3:**
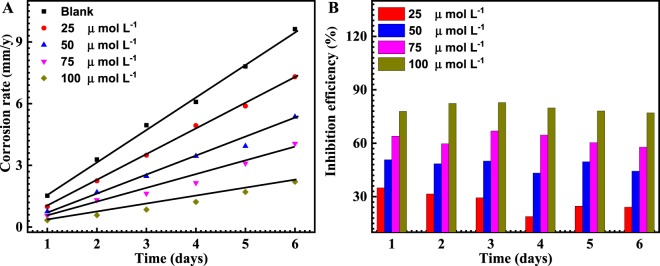
Corrosion rate (**A**), inhibition efficiency (**B**) curves of API X120 steel in carbonated 3.5 wt% NaCl solution with 0, 25, 50, 75 and 100 µmol L^−1^ of PEI corrosion inhibitor at room temperature.

Figure 3 represents the corrosion rate and the inhibition efficiency analysis of API X120 steel immersed in 3.5 wt% NaCl solution saturated with CO_2_ without and with (25, 50, 75 and 100 μmol L^−1^) of PEI corrosion inhibitor for different exposure time at room temperature. The depicted figures were derived from the weight loss measurement as the corrosion rate (*CR*) was calculated according to the equations below^41^.

1 $$CR({\rm{mm}}/{\rm{y}})=\frac{87.6\ast W}{D\ast A\ast t}$$

where *w* is mass loss in mg, *ρ* is the carbon steel density in g/cm^3^, *A* is the surface area of sample in square and *t* is time of test in hours

The inhibition efficiency (*IE*%) and the surface coverage θ, of the inhibitor for the corrosion of HSLA steel was computed as follows,

2 $$IE \% =\theta \times 100=\frac{({W}^{O}-W)}{{W}^{O}}\times 100$$

where *w*° and *w* are the average weight loss values without and with adding the inhibitor, respectively.

It could be noticed from Fig. 3A that the corrosion rate decreases as the PEI corrosion inhibitor concentration increases, suggesting that a greater number of PEI molecules are adsorbed over the active sites of the metal surface hence diminishing the direct contact between the API X120 steel and the corrosive environment^31,42^. Additionally, Fig. 3B shows that the inhibition efficiency percentage increase with raising the concentration of PEI. This is attributed to the development of coordination bond because of overlap of the lone pair electron of the nitrogen atom with the 3d orbital of the iron atom with increasing the inhibition abilities of the PEI molecules on the metal surface^38,43^.

### Potentiodynamic polarization analysis

**Figure 4:**
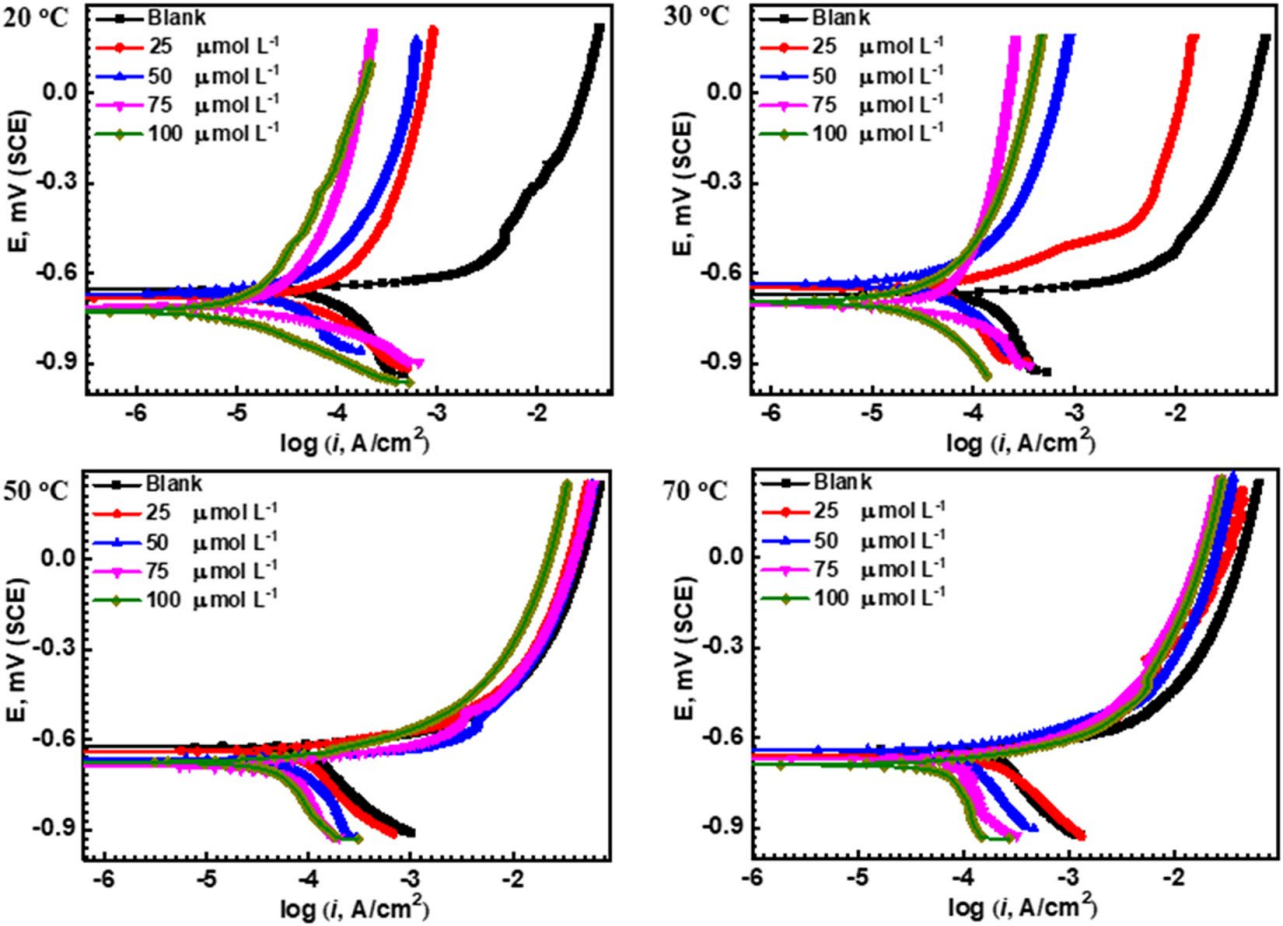
Potentiodynamic polarization graphs for API X120 steel at 0.167 mV S^−1^ scan rate in carbonated 3.5 wt% NaCl solution in the presence and the absence of 25, 50, 75 and 100 mol L^−1^ of PEI at different temperatures where (**A**) 20, (**B**) 30, (**C**) 50 and (**D**) 70 °C.

Figure 4 describes the potentiodynamic polarization curves of HSAL steel in 3.5 wt% NaCl solution saturated with CO_2_ having various concentrations of Polyethyleneimine (PEI) from 20 to 70 °C. The electrochemical kinetic parameters include corrosion free potential (*E*_corr_), corrosion current density (*i*_corr_), the polarization resistance, (*R*_p_) and cathodic/anodic Tafel slopes (*ß*_c_ and *ß*_a_, respectively) were measured by Tafel extrapolation of the current - potential lines to the corresponding corrosion potentials. The measured data is listed in Table 2. Moreover, the corrosion rates were calculated considering the whole surface of API X120 steel is attacked by the aggressive media without any localized corrosion^44^.

3 $$corrosion\,rate\,(mpy)=\frac{0.13\ast {i}_{{\rm{corr}}}\ast A}{n\ast D}$$

where 0.13 is the measurement of the time conversion factor, *i*_corr_ is the corrosion current density (A. cm^−2^), *A* is the atomic weight of iron (55.6 g. mol^−1^), *n* is the number of transferred electrons per metal atom, *D* is the density of iron (7.85 g. cm^−3^),

**Table 2:** Potentiodynamic polarization parameters for API X120 steel in 3.5 wt% NaCl solution with CO_2_ purging in the presence and the absence of different concentration of PEI corrosion inhibitor under elevated temperature.

*T, °C*	*C*_inh_ µmol L^−1^	^E^_corr_ (mV) SCE	*i*_corr_ (µAcm^−2^)	β_a,_ mV/decade	βc, mV/decade	*CR*, *mpy*	*θ*	IE, %
20	Blank	−700	81	82.10	347.8	47.04	—	—
	25	−610	36.3	812.2	9089	21.82	0.55	55.18
	50	−642	28.8	1329	781.1	16.73	0.64	64.44
	75	−670	19.3	64.0	910.9	11.25	0.76	76.17
	100	−577	10.9	730.7	201.5	6.35	0.86	86.54
30	Blank	−661	96.9	4209	5111	56.28	—	—
	25	−662	47	80.5	341.5	27.29	0.51	51.49
	50	−690	39.5	84.4	1781	22.94	0.59	59.23
	75	−624	29	64.40	579.6	16.85	0.70	70.07
	100	−664	18.1	54.4	573.8	12.85	0.81	81.32
50	Blank	−638	121	2664	321.4	70.25	—	—
	25	−700	82.9	82.2	5279	38.82	0.31	31.71
	50	−674	68.3	2060	1890	33.89	0.43	43.55
	75	−641	47	72.20	528.3	27.21	0.61	61.15
	100	−675	35.3	105.8	592.6	20.59	0.70	70.82
70	Blank	−680	186	129.7	4389	108.2	—	—
	25	−638	147	107.4	703.8	85.38	0.20	20.96
	50	−653	120.5	1081	158.9	69.985	0.35	35.21
	75	−624	88	71	432.9	51.16	0.52	52.68
	100	−637	64	253.2	9583	37.16	0.65	65.59

The surface coverage values (*θ*) was obtained from the following equation^45^:

4 $$\theta =\frac{{i}_{corr}^{o}-{i}_{{\rm{corr}}}}{{i}_{{\rm{corr}}}}$$

where *i°*_corr_ and *i*_*corr*_ are the corrosion current densities of API X120 steel without and with the presence of the corrosion inhibitor, respectively. Moreover, the corrosion inhibition efficiency (*IE*%) is calculated using Eq. 5.

5 $$IE \% =\theta \times 100$$

It is worth noting that, the potentiodynamic polarization curves of API X120 steel in 3.5 wt% NaCl solution saturated with CO_2_ shows increase in cathodic and anodic current densities with increasing temperature. This is attributed to the hydrogen reduction reaction in the cathodic region and the early dissolution of API X120 steel in the anodic part^46^. Meanwhile, in the presence of PEI corrosion inhibitor, with increasing amount of PEI concentration the cathodic and the anodic current densities are shifted to lower values when compared to the blank. Furthermore, the corrosion potential is slightly shifted to the positive direction. It is reported that if the difference between the *E*_corr_ value of inhibitor and the *E*_corr_ value of the aggressive media is more than ±85 mV, the inhibitor will be either cathodic type or anodic, otherwise the corrosion inhibitor will be classified as a mixed type inhibitor. The *E*_corr_ values in Table 2 confirms that the PEI is a mixed type inhibitor and it has a significant effect in reducing the corrosion rates (mpy) compared to the blank. The higher the concentration of the PEI, the lower are the corrosion rates at all studied temperatures. However, it is further noticed that the increase in temperature increases the corrosion rate at the same concentration of the PEI^47,48^.

### Electrochemical impedance spectroscopic analysis

**Figure 5:**
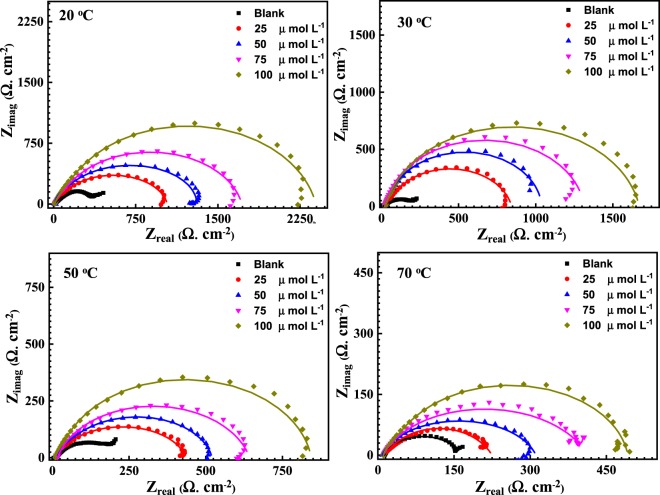
EIS measured Nyquist spectra (dotted) and their fitted curves (solid lines) for API X120 steel in carbonated 3.5 wt% NaCl solution with 0, 25, 50, 75 and 100 µmol L^−1^ of PEI corrosion inhibitor at (**A**) 20, (**B**) 30, (**C**) 50 and (**D**) 70 °C.

Figure 5 represents the EIS results for API X120 steel specimens in 3.5 wt% NaCl solution saturated with CO_2_ containing different concentrations of PEI (0, 25, 50, 75 and 100 µmol L^−1^) conducted at various temperatures. Clearly, the diameters of the semicircles of the Nyquist graphs of the PEI inhibitor at concentrations of 25, 50, 75 and 100 µmol L^−1^ are larger compared to the blank one. It is also clear the diameters of the semicircles decrease as the temperature increase at the same concentration of the PEI. In addition, the depressed capacitive loops seen at low frequency in all the Nyquist plots refer to a charge transfer mechanism for the corrosion of API X120 steel in 3.5 wt% NaCl solution saturated with CO_2_. The deviation of the capacitive loop from a complete semi-circle might be because of the heterogeneity and microroughness of the working electrode surface^49,50^.

**Figure 6:**
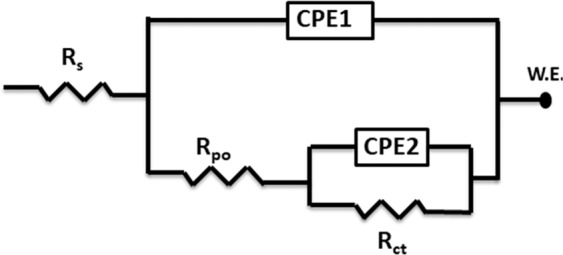
An electrical equivalent circuit used for the analysis of EIS measured data.

Figure 6 shows the electrical equivalent circuit (EC) that is used for the EIS analysis. It is a two-time constant equivalent circuit in parallel type which commonly used to describe a non-uniform corrosion of electrodes in an electrolyte^51,52^. The EC contains uncompensated solution resistance (*R*_s_), a pore resistance (*R*_po_), a charge transfer resistance (*R*_ct_) and two constant phase elements (*CPE1, CPE2*) that replaces the capacitive element to obtain a more accurate fit. The non-ideal layer capacitors are predictable in the recorded double layer capacitances. This can be attributed to many reasons such as the non-uniformity and the surface roughness of the tested sample, the current distribution of the inhibitor or the corrosion products, the surface coverage and the corrosion rate^53,54^.

The impedance of the *CPE* is expressed by Eq. 6^55,56^:

6 $${Z}_{Q}=[{Y}_{o}^{-1}{(j\omega )}^{-n}]$$

where *Z*_Q_ is the *CPE* impedance value (Ω cm^−2^); ${Y}_{o}$ is the *CPE* constant; $j$ = (−1)^1/2^ which apparently equal to the imaginary number; $\omega$ = 2π*f*_max_ is the angular frequency in rad/s and *f*_max_ the maximum frequency for the imaginary part; n is the fitting roughness and its values between 0 and 1. When n = 0, the *CPE* becomes equivalent to a resistor and when n = 1, the *CPE* becomes equivalent to an ideal capacitor. All EIS parameters obtained from the Nyquist graphs are summarized in Table 3.

**Table 3:** Dynamic Electrochemical Impedance Parameters for API X120 steel in 3.5 wt% NaCl solution without and with Co_2_ purging in the absence and presence of various concentrations of PEI corrosion inhibitor.

T, °C	*C*_inh_ µmol L^−1^	*R*_s_,	*R*_po_,	*Y*_po_ × 10^−6^ s^n^*Ω* ^−1^ cm^−2^	n_1_	*R*_*ct*_,	*Y*_ct_ × 10^−6^ s^n^*Ω* ^−1^ cm^−2^	n_2_	*θ*	*IE, %*
20	Blank	12.8	62.02	154.4	0.904	345.1	119.27	0.880	—	—
	25	12.2	107.4	153.1	0.906	1082	68.19	0.965	0.681	68.10
	50	11.4	187.4	127.5	0.886	1398	63.19	0.968	0.753	75.31
	75	13.6	274.4	126.6	0.918	1725	61.85	0.974	0.799	79.99
	100	13.29	136.7	113.9	0.884	2416	49.6	0.865	0.857	85.71
30	Blank	14.17	66.82	286.5	0.985	308	296.4	0.857	—	—
	25	14.2	19.63	231.1	0.735	822	254.1	0.849	0.625	62.53
	50	13.5	31.6	223.3	0.839	1042	217.8	0.884	0.704	70.44
	75	11.18	36.09	141.7	0.891	1245	166.6	0.974	0.752	75.26
	100	12.6	40.6	123.6	0.813	1715	102.7	0.836	0.820	82.04
50	Blank	13.05	21.05	306.5	0.722	242.7	550.1	0.734	—	—
	25	9.35	14.5	250.3	0.752	428	384.6	0.838	0.432	43.29
	50	11.7	44.4	225.6	0.956	532	323.9	0.965	0.543	54.37
	75	11.9	20.9	165.1	0.913	662	291.6	0.946	0.633	63.33
	100	11.03	29.4	137.17	0.647	858	288.1	0.829	0.717	71.71
70	Blank	11.14	23.5	320.6	0.608	180	858.8	0.846	—	—
	25	12.4	36.4	299.3	0.678	242.7	625.1	0.966	0.258	25.83
	50	12.7	47.4	249.6	0.914	313.2	570.1	0.530	0.425	42.52
	75	13.68	51.74	223.8	0.811	397.2	569.2	0.986	0.546	54.68
	100	12.39	55.4	222.7	0.592	504.2	532.5	0.986	0.642	64.29

The surface coverage (*θ*) is estimated using the following equation^31,57^:

7 $$\theta =\frac{{R}_{{\rm{ct}}1}-{R}_{{\rm{ct}}2}}{{R}_{{\rm{ct}}1}}$$

where *R*_ct1_and *R*_ct2_ are the charge-transfer resistances in the absence and the presence of the PEI corrosion inhibitor, respectively. Moreover, the corrosion inhibition efficiency (*IE*%) was calculated using Eq. 3.

It can be noticed that the API X120 steel surface is more corrosion resistant with 100 μmol L^−1^ of PEI than the lower concentrations of PEI in a 3.5% NaCl saturated CO_2_ purging solution. *R*_ct_ increases while the *C*_dl_ decreases with increasing the PEI concentration or decreasing the temperature^58^. For instance, comparing the blank solution with the one containing 25 μmol L^−1^ of PEI at 20 °C, the *R*_ct_ increases from 345 Ω cm^2^ to 1082 Ω cm^2^, and *C*_dl_ decreases from 119.27 µF to 68.75 µF with an inhibition efficiency of 68.4%. Meanwhile, increasing the inhibitor concentration to 100 μmol L^−1^, increases the *R*_ct_ value to 2416 Ω cm^2^, and the *C*_dl_ decreases to 49.51 µF with an inhibition efficiency of 85.6%. This observation would be explained by the Helmholtz equation as expressed below^59^.

8 $${\delta }_{ads}=\frac{{\rm{\varepsilon }}\,{\rm{\varepsilon }}{\rm{o}}\,A}{{C}_{{\rm{dl}}}}$$

where *δ*_ads_ is the thickness of the PEI corrosion inhibitor adsorbed layer, ε_o_ is the air permittivity, *ε* is the local dielectric constant and *A* is the area of API X120 steel electrode. This equation shows *C*_dl_ is inversely proportional to *δ*_ads_ i.e. the decrease of the *C*_dl_ value is attributed to the growth of the adsorbed film of PEI corrosion inhibitor as its concentration increases in solution. As the protective layer increase, the charge transfer become more sluggish as shown from the *R*_ct_ and *IE*% values. Increasing the temperature will promote the desorption rate of the PEI molecules from the API X120 steel surface and raise the dissolution rate of the Fe ions which lead to decrease in the inhibition efficiency. Generally, increasing the PEI concentration shift the “n” values to the less positive direction. This make the constant phase element becoming farther from the ideal capacitor^60,61^. It is worthy to mention that the EIS measurements are in agreement with the potentiodynamic polarization data.

### Inhibitor adsorption and thermodynamic analysis

**Figure 7:**
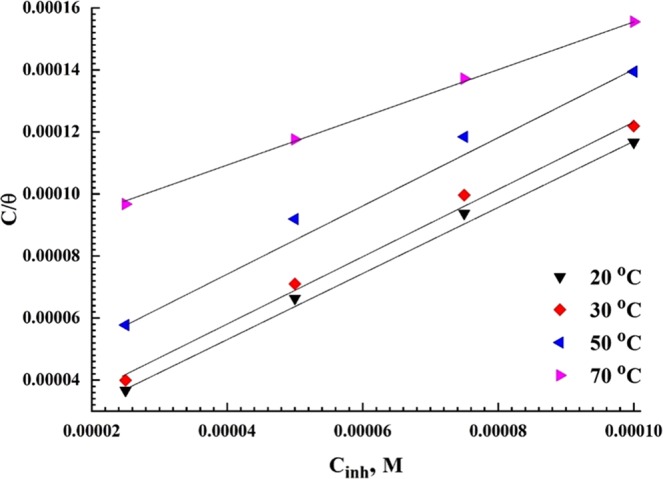
Langmuir adsorption plots for API X120 steel in carbonated 3.5 wt% NaCl with saturated CO_2_ solution under elevated temperature.

The reaction of metal active sites with the corrosion inhibitor molecules occurs via substitutional replacement of the electrolyte molecules at metal/solution interface^62–64^. The adsorption isotherm models determine the type of reaction i.e. whether it is spontaneously or non-spontaneously and whether the interaction is physical or chemical. Figure 7 shows the relation between $\frac{{C}_{inh}}{\theta }$ and *C*_*inh*_ at different temperatures according to Langmuir adsorption isotherm equation^65^.

9 $$\frac{\theta }{1-\theta }={K}_{{\rm{ads}}}\ast {C}_{{\rm{inh}}}$$

where *K*_ads_ is the equilibrium constant of the adsorption-desorption process, and *C*_inh,_ is the inhibitor concentration. Straight lines are obtained with a slope close to 1, with a correlation coefficient (R^2^ > 0.99). The values of *K*_ads_ are calculated from the intercepts of the plotted straight lines with y-axis.

The standard free energy of adsorption reaction, *ΔG°*_*ads*_, in kJ mol^−1^, is calculated using *K*_ads_ values from the following equation^66^:

10 $${K}_{ads}=\frac{1}{55}\exp \,(\frac{-\Delta {G}_{ads}^{o}}{RT})$$

where *R* is the universal gas constant in J mol^−1^ K^−1^ and *T* is the absolute temperature. The values of *K*_*ads*_ and *ΔG°*_*ads*_, are given in Table 4. The higher *K*_ads_ value indicates the strong adsorption ability of the PEI inhibitor. In addition, the calculated *ΔG°*_*ads*_ values of the used PEI corrosion inhibitor is close to −40 kJ mol^−1^ at 20 °C and decreases as the temperature increases. Thus, it can be assumed that the PEI molecules adsorbed on the metal surface and demonstrate acceptable desorption properties in the polarization graphs. Finally, the adsorption mechanism of the PEI molecules in the API X120 steel surface in CO_2_ saturated solution is followed a mixed physi-chemisorption mechanism^67,68^.

**Table 4:** Thermodynamic Parameters derived from the Langmuir plots under elevated temperature.

Temperature, K	*K*_ads_	*R*^2^	*∆G*°_ads_ (kJ mol^−1^)
293.15	86.3	0.995	−37.5
303.15	68.8	0.998	−38.2
323.15	29.4	0.990	−38.4
343.15	12.9	0.994	−38.4

### Thermodynamic activation parameters and inhibition mechanism

**Figure 8:**
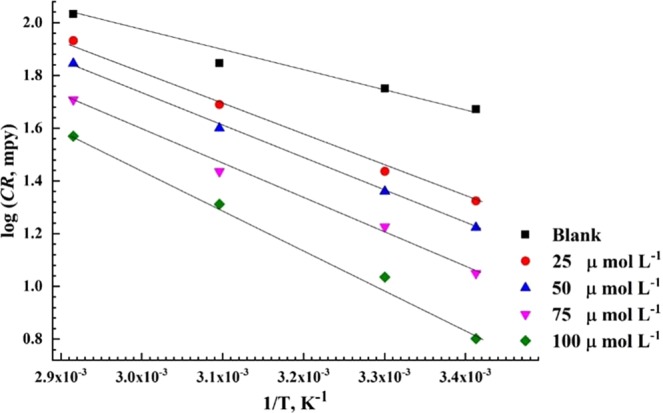
Arrhenius graph derived from corrosion rate logarithmic versus reciprocal of absolute temperature at different concentration of PEI corrosion inhibitor.

To estimate the activation energy (*E*_a_) for the corrosion of API X120 steel in 3.5 wt% NaCl solution saturated with CO_2_ in diverse concentrations of PEI corrosion inhibitor at temperature range of 20 °C to 70 °C, the logarithm of the corrosion rate (CR) which can be expressed by log (*CR*) was plotted against 1/*T* according to Arrhenius equation^69^:

11 $$CR=A\,\exp \,(\frac{-{E}_{a}}{RT})$$

where, A is the Arrhenius constant that depends on the metal type and electrolyte^70^. The *E*_a_ values calculated from the slopes of the plotted straight lines in Fig. 8 that have high regression coefficient close to unity are listed in Table 5. The addition of PEI corrosion inhibitor increases the activation energy value which indicate a strong physical adsorption of PEI compound on API X120 surface.

**Table 5:** Thermodynamic activation parameters derived from the Arrhenius and transition state equations for each concentration.

Conc. of inhibitor µmol L^−1^	*E*_a_(kJ mol^−1^)	ΔH^*^ (kJ. Mol^−1^)	ΔS^*^ (J. mol^−1^ K^−1^)
0	−13.3	−10.69	−176.3
25	−23.5	−20.77	−149.8
50	−23.7	−21.13	−149.2
75	−24.5	−26.24	−148.7
100	−28.9	−21.91	−139.4

However, the adsorption of PEI molecules on API X120 steel as shown $\Delta {G}_{ads}^{o}$ occurs through a simultaneous physi/chemisorption process. The activation energy solely would not elucidate the type of the adsorption because there is a rivalry between the PEI corrosion inhibitor and the OH^−^ group from water molecules for adsorbing on the metal surface and for removing the OH^−^ group away from the metal surface and thus extra activation energy would be required^71,72^. According to the transition state equation, the apparent enthalpy of activation, Δ*H*_a_, and entropy of activation, Δ*S*_a_, for API X120 corrosion in 3.5 wt% NaCl solution saturated with CO_2_ can be calculated from the corrosion rate (corrosion current densities) at different temperatures in the presence and the absence of various concentrations of the PEI corrosion inhibitor^73,74^.

12 $$CR=(\frac{RT}{{N}_{A}h})\exp \,(\frac{\Delta {S}_{a}}{R\,})\exp (\frac{-\Delta {H}_{a}}{RT\,})$$

**Figure 9:**
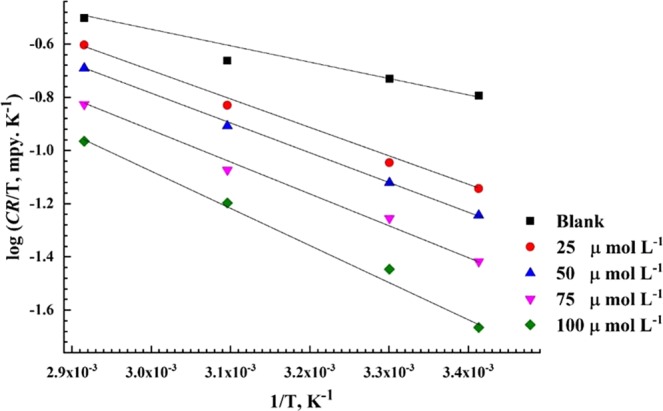
Transition-state plots of log (*CR/T*) versus 1/*T* for API X120 steel in solution in absence and presence of different concentrations of PEI corrosion inhibitor.

*“h”* is the Planck’s constant, *N*_*A*_, is the Avogadro’s number, and *R*, is the universal gas constant. Plotting log (*CR*/*T*) against 1*/T* yield straight lines relation as shown in Fig. 9.

The values of ΔH^*^ and ΔS^*^ are calculated from the slopes of the straight lines and their intercepts with the y-axis, respectively and are tabulated in Table 5. The endothermic nature of the API X120 dissolution reaction is inferred from the positive sign of ΔH^*^. Increasing the ΔH^*^ values as the concentration of inhibitor increase means that the dissolution of API X120 becomes more sluggish in presence of the PEI inhibitor^75^. The negative values of ΔS^*^confirms that the activated complex is the rate determining step where association rather than dissociation take place as the reaction goes from the reactants to the activated complex step^76^.

### AFM investigation

**Figure 10:**
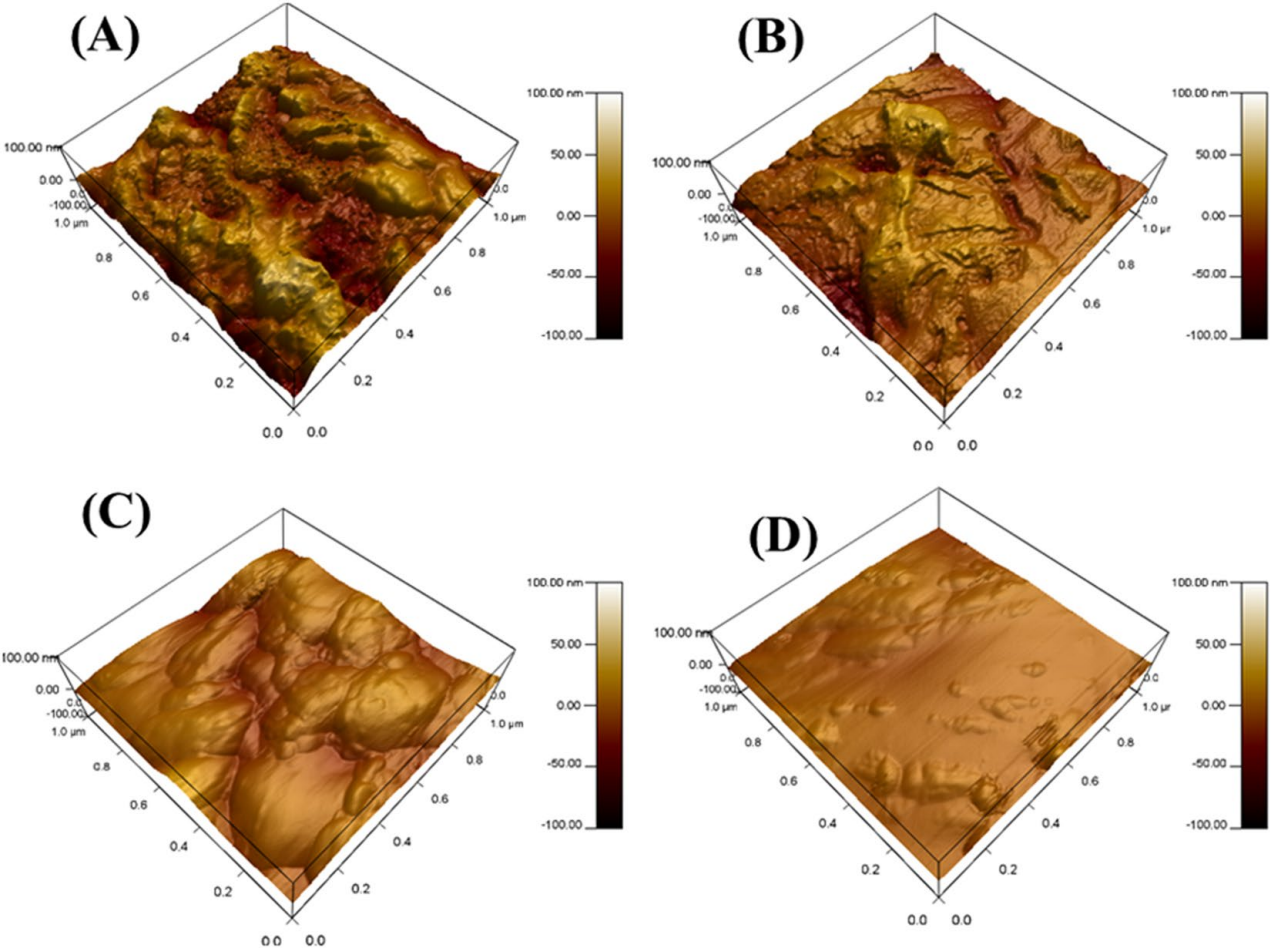
AFM images for API X120 steel surface after immersion for 6 hr. In (**A**) 3.5 wt% NaCl solution, (**B**) CO_2_ saturated brine solution (**C**) 3.5 wt% NaCl with 100 µmol L^−1^ PEI solution and (**D**) CO_2_ saturated brine solution with 100 µmol L^−1^ PEI at 20 °C.

Figure 10(A) shows the surface analysis of API-X120 steel after its immersion in 3.5 wt% NaCl for 6 h at room temperature, where a severely corroded surface is observed with mean roughness value (*R*_a_) of 460 nm. Meanwhile, the peaks and the valleys heights reduced to 211 nm after adding 100 μmol L^−1^ of PEI as seen in Fig. 10(B). On the other hand, Fig. 10(C,D) represent the 3D surface topography images of API X120 steel samples immersed in 3.5 wt% NaCl solution saturated with CO_2_ in the absence and the presence of 100 μmol L^−1^ of PEI, respectively. It can be noticed that there is less damage in comparison with the samples without CO_2_ purging. The R_a_ values also reduced from 230 to 131 nm as seen in Fig. 10(C,D) respectively. This is attributed to the affinity of the PEI molecules to be adsorbed on the metal surface through heteroatoms and formed a protective layer which reduces the corrosion rate and the surface roughness^77^. This is attributed to the compact protective film limits the aggressive ions transfer towards the metallic surface and reduces the corrosion rate.

### Scanning electron microscopic analysis

**Figure 11:**
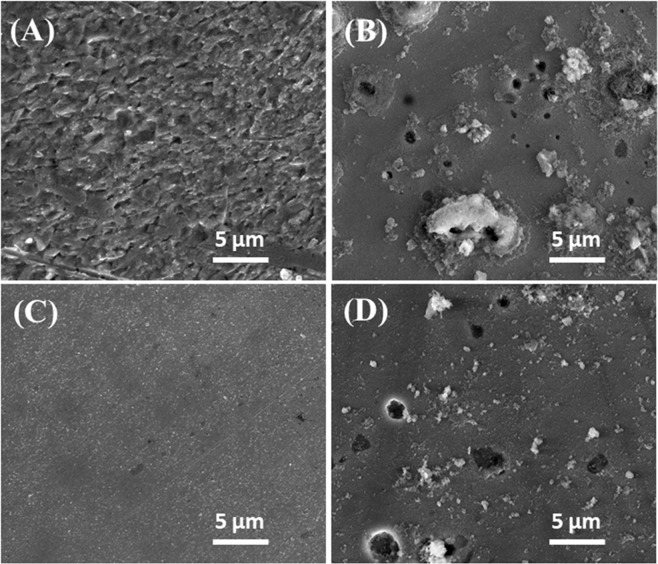
SEM micrographs for API X120 steel surface after immersion for 6 hr. In (**A**) 3.5 wt% NaCl solution, (**B**) CO_2_ saturated brine solution (**C**) 3.5 wt% NaCl with 100 µmol L^−1^ PEI solution and (**D**) CO_2_ saturated brine solution with 100 µmol L^−1^ PEI at 20 ^o^C.

**Figure 12:**
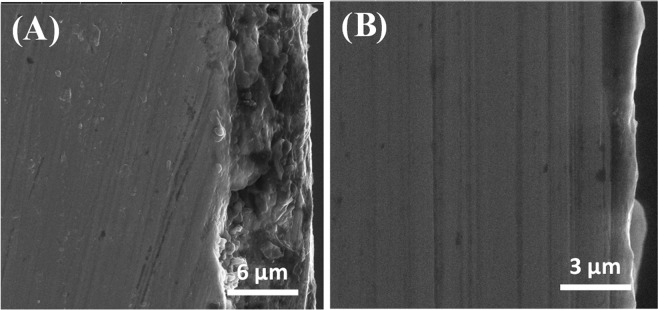
Cross sectional SEM micrographs for API X120 steel surface after immersion for 24 hr. in CO_2_ saturated brine solution (**A**) in absence and (**B**) presence 100 µmol L^−1^ PEI at 20 ^o^C.

The surface morphology of API X120 steel when exposed to various aggressive media after 6 hrs of immersion at 20 °C is presented in Fig. 11. Steel surface is extremely corroded and roughened due to a highly aggressive 3.5 wt% NaCl solution with a lot of clear pits as seen in Fig. 11(A). Figure 11(B) depicts the specimen’s surface in the presence of the 100 µmol L^−1^ of PEI in 3.5 wt% NaCl solution. It can be noticed that only a little number of pits can be observed. Figure 11(C) shows a dense corrosion product scale formed in CO_2_ saturated brine solution. Meanwhile, in Fig. 11(D) the scale corrosion product influenced further diminished due to the presence of 100 µmol L^−1^ of PEI to the brine solution, which decreases the steel surface roughness. This indicates that although the scale (corrosion product-siderite) seems to provide some corrosion protection to the metal surface, the PEI molecules have the ability to prohibit the scale formation and increase the corrosion protection due to capturing the CO_2_ from the brine solution as shown in Fig. 12. The cross-sectional examination shows that the thickness of the formed layer is significantly diminished in favor of adding 100 μmol L^−1^ of the PEI corrosion inhibitor as it drops from 7 μm to 900 nm in the absence and the presence of 100 μmol L^−1^ of the PEI, respectively.

### Corrosion inhibition mechanism

PEI is a branched water-soluble macromolecule polymer and there are a large number of amine groups on it. Each nitrogen atom in amine group contains a lone pair of electrons which provides a strong electron donating affinity to PEI molecule. It is clear that PEI has corrosion inhibition properties towards API X120 steel in neutral media. This is due to the electrostatic interaction between the positive ions on the metal surface and the partially negative charge PEI molecules. Consequently, PEI molecule adsorbs on the steel surface physically and 100 μmol L^−1^ of PEI gives 88% corrosion inhibition efficiency. On the other hand, the PEI molecule has a CO_2_ sorption affinity which influence the CO_2_ molecule to interact with the PEI adsorbed molecule instead of the metal surface^78–83^. As a result, a dense protective layer formed over the metal surface in the presence of the PEI corrosion inhibitor and it is more compact than the corresponding layer with the CO_2_ as seen in Fig. 12. Therefore, the complex PEI film on the steel surface act as a barrier which hinder the aggressive ions to penetrate to the steel surface as well as prohibiting the Fe dissolution^38,84–87^.

## Conclusions

The PEI can efficiently inhibit the corrosion of API X120 steel (API X120) in 3.5 wt% NaCl solution saturated with CO_2_ as it builds a protective film from adsorbed molecules on the metal surface. PEI molecules exhibit a strong affinity for metal surface therefore shows good inhibition efficiency. The inhibition efficiency of API X120 steel increases with increasing concentration of PEI in 3.5 wt% NaCl solution saturated with CO_2_. Potentiodynamic polarization measurements for APIX 120 steel immersed in CO_2_ saturated saline solution confirm that PEI functions as a mixed type corrosion inhibitor as the cathodic and the anodic reaction rate has been reduced. The PEI molecules are exothermically mixed physi/chemisorbed on the API X120 steel surface, as confirmed from the calculated standard Gibbs free energy change ($\Delta {G}_{ads}^{o})$. SEM and AFM images depict that the surface roughness of the exposed steel to CO_2_ saturated saline solution is significantly higher than that in the corresponding PEI solution.

## Data Availability

The raw data required to reproduce these findings can be shared at any time based on direct requests to the authors.

## References

[1] Herrán A, de la Cruz JM, de Andrés B (2012). Global Search Metaheuristics for planning transportation of multiple petroleum products in a multi-pipeline system. Computers & Chemical Engineering.

[2] Carretero Olalla V (2014). Analysis of the strengthening mechanisms in pipeline steels as a function of the hot rolling parameters. Mater. Sci. Eng., A.

[3] Okonkwo PC, Shakoor RA, Ahmed E, Mohamed AMA (2016). Erosive wear performance of API X42 pipeline steel. Eng. Fail. Anal..

[4] Shakoor, A. *et al*. Corrosion behavior of high strength low alloy HSLA steel in 35 wt% NaCl solution containing diethylenetriamine DETA as corrosion inhibitor. **2018**, 10.5339/qfarc.2018.EEPD356 (2018).

[5] Ríos-Mercado RZ, Borraz-Sánchez C (2015). Optimization problems in natural gas transportation systems: A state-of-the-art review. Appl. Energy.

[6] Guo Y, Meng T, Wang D, Tan H, He R (2017). Experimental research on the corrosion of X series pipeline steels under alternating current interference. Eng. Fail. Anal..

[7] Mohtadi-Bonab MA, Eskandari M (2017). A focus on different factors affecting hydrogen induced cracking in oil and natural gas pipeline steel. Eng. Fail. Anal..

[8] Pedrosa IRV, Castro RSd, Yadava YP, Ferreira RAS (2013). Study of phase transformations In API 5L X80 Steel in order to increase its fracture toughness. Mater. Res..

[9] Zhang J-m, Sun W-h, Sun H (2010). Mechanical Properties and Microstructure of X120 Grade High Strength Pipeline Steel. J. Iron. Steel Res. Int..

[10] Cao R (2018). The effects of Silicon and Copper on microstructures, tensile and Charpy properties of weld metals by refined X120 wire. Mater. Sci. Eng., A.

[11] Yoo J-Y, Ahn S-S, Seo D-H, Song W-H, Kang K-B (2011). New Development of High Grade X80 to X120 Pipeline Steels. Mater. Manuf. Processes.

[12] Linter BR, Burstein GT (1999). Reactions of pipeline steels in carbon dioxide solutions. Corrosion Sci..

[13] Gao M, Pang X, Gao K (2011). The growth mechanism of CO2 corrosion product films. Corrosion Sci..

[14] Zhang J, Wang J, Zhu F, Du M (2015). Investigation of Inhibition Properties of Sophorolipids for X65 Steel Corrosion in Simulated Oilfield Produced Water Saturated with Carbon Dioxide. Ind. Eng. Chem. Res..

[15] Mazumder MAJ, Nazal MK, Faiz M, Ali SA (2016). Imidazolines containing single-, twin- and triple-tailed hydrophobes and hydrophilic pendants (CH2CH2NH)nH as inhibitors of mild steel corrosion in CO2–0.5 M NaCl. RSC Adv..

[16] Nešić S (2007). Key issues related to modelling of internal corrosion of oil and gas pipelines – A review. Corrosion Sci..

[17] Kermani MB, Morshed A (2003). Carbon dioxide corrosion in oil and gas production - A compendium. Corrosion.

[18] Ren C, Wang W, Jin X, Liu L, Shi T (2015). Physicochemical performance of FeCO3 films influenced by anions. RSC Adv..

[19] Eliyan FF, Alfantazi A (2013). Influence of temperature on the corrosion behavior of API-X100 pipeline steel in 1-bar CO2-HCO3− solutions: An electrochemical study. Mater. Chem. Phys..

[20] Eliyan FF, Mohammadi F, Alfantazi A (2012). An electrochemical investigation on the effect of the chloride content on CO2 corrosion of API-X100 steel. Corrosion Sci..

[21] Al-Jaroudi SS, Ul-Hamid A, Al-Moumen MA (2015). Premature failure of tubing used in sweet Extra Arab Light grade crude oil production well. Eng. Fail. Anal..

[22] Hernández-Espejel A, Domínguez-Crespo MA, Cabrera-Sierra R, Rodríguez-Meneses C, Arce-Estrada EM (2010). Investigations of corrosion films formed on API-X52 pipeline steel in acid sour media. Corrosion Sci..

[23] Mustafa AH, Ari-Wahjoedi B, Ismail MC (2013). Inhibition of CO2 Corrosion of X52 Steel by Imidazoline-Based Inhibitor in High Pressure CO2-Water Environment. J. Mater. Eng. Perform..

[24] Valcarce MB, Vázquez M (2009). Carbon steel passivity examined in solutions with a low degree of carbonation: The effect of chloride and nitrite ions. Mater. Chem. Phys..

[25] Wang B, Xu L, Liu G, Lu M (2018). Corrosion behavior and mechanism of 3Cr steel in CO2 environment with various Ca2+ concentration. Corrosion Sci..

[26] Umoren S, Ebenso E, Okafor P, Ogbobe O (2006). Water-soluble polymers as corrosion inhibitors. Pigment & Resin Technology.

[27] Ingham B, Ko M, Laycock N, Kirby NM, Williams DE (2015). First stages of siderite crystallisation during CO2 corrosion of steel evaluated using *in situ* synchrotron small- and wide-angle X-ray scattering. Faraday Discuss..

[28] Zheng LF, Landon J, Koebcke NC, Chandan P, Liu KL (2015). Suitability and Stability of 2-Mercaptobenzimidazole as a Corrosion Inhibitor in a Post-Combustion CO2 Capture System. Corrosion.

[29] Arthur DE, Jonathan A, Ameh PO, Anya C (2013). A review on the assessment of polymeric materials used as corrosion inhibitor of metals and alloys. Int. J. Ind. Chem..

[30] Kahraman R (2002). Inhibition of atmospheric corrosion of mild steel by sodium benzoate treatment. J. Mater. Eng. Perform..

[31] Finšgar M, Fassbender S, Nicolini F, Milošev I (2009). Polyethyleneimine as a corrosion inhibitor for ASTM 420 stainless steel in near-neutral saline media. Corrosion Sci..

[32] Finšgar M, Fassbender S, Hirth S, Milošev I (2009). Electrochemical and XPS study of polyethyleneimines of different molecular sizes as corrosion inhibitors for AISI 430 stainless steel in near-neutral chloride media. Mater. Chem. Phys..

[33] Beaglehole D, Webster B, Werner S (1998). Ellipsometry Study of the Adsorption of Molecules at Electrolyte Interfaces with Gold and Stainless Steel. J. Colloid Interface Sci..

[34] Kazazi M, Afshar A, Sajjadnejad M (2013). The Inhibition Effect of Polyethylenimine (PEI) on Pitting Corrosion of 304 Austenitic Stainless Steel in 3.5% NaCl Solution. International Journal of Iron & Steel Society of Iran.

[35] Jianguo Y, Lin W, Otieno-Alego V, Schweinsberg DP (1995). Polyvinylpyrrolidone and polyethylenimine as inhibitors for the corrosion of a low carbon steel in phosphoric acid. Corrosion Sci..

[36] Sekine I (1992). Corrosion Inhibition of Mild Steel by Cationic and Anionic Polymers in Cooling Water System. J. Electrochem. Soc..

[37] Zhang X (2009). Anticorrosion Behaviors of Quaternary Polyethyleneimine in Acidic Environment. Mater. Sci. Forum.

[38] Gao B, Zhang X, Sheng Y (2008). Studies on preparing and corrosion inhibition behaviour of quaternized polyethyleneimine for low carbon steel in sulfuric acid. Mater. Chem. Phys..

[39] Ali SA, Saeed MT (2001). Synthesis and corrosion inhibition study of some 1,6-hexanediamine-based N,N-diallyl quaternary ammonium salts and their polymers. Polymer.

[40] G1-90-e1, A. Standard Practice for Preparing, Cleaning, and Evaluating Corrosion Test Specimens. (1999).

[41] Singh A (2017). An impending inhibitor useful for the oil and gas production industry: Weight loss, electrochemical, surface and quantum chemical calculation. Scientific Reports.

[42] Sliem MH (2019). AEO7 Surfactant as an Eco-Friendly Corrosion Inhibitor for Carbon Steel in HCl solution. Scientific Reports.

[43] Salah M, Lahcène L, Omar A, Yahia H (2017). Study of corrosion inhibition of C38 steel in 1 M HCl solution by polyethyleneiminemethylene phosphonic acid. International Journal of Industrial Chemistry.

[44] Yaqo EA, Anaee RA, Abdulmajeed MH, Tomi IHR, Kadhim MM (2020). Potentiodynamic polarization, surface analyses and computational studies of a 1,3,4-thiadiazole compound as a corrosion inhibitor for Iraqi kerosene tanks. J. Mol. Struct..

[45] Ortega-Toledo DM, Gonzalez-Rodriguez JG, Casales M, Martinez L, Martinez-Villafañe A (2011). CO2 corrosion inhibition of X-120 pipeline steel by a modified imidazoline under flow conditions. Corrosion Sci..

[46] Zhao J, Chen G (2012). The synergistic inhibition effect of oleic-based imidazoline and sodium benzoate on mild steel corrosion in a CO_2_-saturated brine solution. Electrochimi. Acta.

[47] Schweinsberg DP, Hope GA, Trueman A, Otieno-Alego V (1996). An electrochemical and SERS study of the action of polyvinylpyrrolidone and polyethylenimine as inhibitors for copper in aerated H_2_SO_4_. Corrosion Sci..

[48] Vashisht H (2016). Synergistic interactions between tetra butyl phosphonium hydroxide and iodide ions on the mild steel surface for corrosion inhibition in acidic medium. J. Mol. Liq..

[49] Bahgat Radwan A, Sliem MH, Okonkwo PC, Shibl MF, Abdullah AM (2017). Corrosion inhibition of API X120 steel in a highly aggressive medium using stearamidopropyl dimethylamine. J. Mol. Liq..

[50] Usman BJ, Umoren SA, Gasem ZM (2017). Inhibition of API 5L X60 steel corrosion in CO2-saturated 3.5% NaCl solution by tannic acid and synergistic effect of KI additive. J. Mol. Liq..

[51] Okonkwo PC, Sliem MH, Shakoor RA, Mohamed AMA, Abdullah AM (2017). Effect of Temperature on the Corrosion Behavior of API X120 Pipeline Steel in H2S Environment. J. Mater. Eng. Perform..

[52] Javidi M, Bekhrad S (2018). Failure analysis of a wet gas pipeline due to localised CO_2_ corrosion. Eng. Fail. Anal..

[53] Singh A (2015). Electrochemical and surface studies of some Porphines as corrosion inhibitor for J55 steel in sweet corrosion environment. Appl. Surf. Sci..

[54] Javidi M, Khodaparast M (2015). Inhibitive Performance of Monoethylene Glycol on CO2 Corrosion of API 5L X52 Steel. J. Mater. Eng. Perform..

[55] Zhang H-h, Pang X, Gao K (2018). Localized CO2 corrosion of carbon steel with different microstructures in brine solutions with an imidazoline-based inhibitor. Appl. Surf. Sci..

[56] Feng L, Yang H, Cui X, Chen D, Li G (2018). Experimental and theoretical investigation on corrosion inhibitive properties of steel rebar by a newly designed environmentally friendly inhibitor formula. RSC Adv..

[57] Saha SK, Dutta A, Ghosh P, Sukul D, Banerjee P (2016). Novel Schiff-base molecules as efficient corrosion inhibitors for mild steel surface in 1 M HCl medium: experimental and theoretical approach. Phys. Chem. Chem. Phys.

[58] Zhang BR (2015). Synergistic corrosion inhibition of environment-friendly inhibitors on the corrosion of carbon steel in soft water. Corrosion Sci..

[59] Mansfeld F (1995). Use of electrochemical impedance spectroscopy for the study of corrosion protection by polymer coatings. J. Appl. Electrochem.

[60] Abd-Elaal AA, Elbasiony NM, Shaban SM, Zaki EG (2018). Studying the corrosion inhibition of some prepared nonionic surfactants based on 3-(4-hydroxyphenyl) propanoic acid and estimating the influence of silver nanoparticles on the surface parameters. J. Mol. Liq..

[61] Gadala IM, Alfantazi A (2014). Electrochemical behavior of API-X100 pipeline steel in NS4, near-neutral, and mildly alkaline pH simulated soil solutions. Corrosion Sci..

[62] Laidler, K. J. React. Kinet., (Pergamon Press, New York, 1963.).

[63] Lukovits I, Kálmán E, Zucchi F (2001). Corrosion Inhibitors—Correlation between Electronic Structure and Efficiency. Corrosion.

[64] Fouda, A. e., Abd El-Aal, A., Sliem, M. H. & Abdullah, A. Caprylamidopropyl Betaine as a highly efficient eco-friendly corrosion inhibitor for API X120 steel in 1 M H2SO4. *Egypt. J. chem*., 10.21608/ejchem.2019.13652.1844 (2019).

[65] Ren X, Xu S, Chen S, Chen N, Zhang S (2015). Experimental and theoretical studies of triisopropanolamine as an inhibitor for aluminum alloy in 3% NaCl solution. RSC Adv..

[66] El-Haddad MN (2014). Hydroxyethylcellulose used as an eco-friendly inhibitor for 1018 c-steel corrosion in 3.5% NaCl solution. Carbohydr. Polym..

[67] Sureshkumar Srinivasan, A. V. A. A. A. Screening and Evaluating Environmentally-Friendly Corrosion Inhibitors for Amine-Based CO2 Absorption Process, Corrosion Inhibitors, Principles and Recent Applications Mahmood Aliofkhazraei. IntechOpen (April 4th 2018).

[68] Espinoza-Vázquez A, Rodríguez-Gómez FJ (2016). Caffeine and nicotine in 3% NaCl solution with CO2 as corrosion inhibitors for low carbon steel. RSC Adv..

[69] Mobin M, Khan MA (2014). Adsorption and Corrosion Inhibition Behavior of Polyethylene Glycol and Surfactants Additives on Mild Steel in H2SO4. J. Mater. Eng. Perform..

[70] Radwan AB, Sliem MH, Yusuf NS, Alnuaimi NA, Abdullah AM (2019). Enhancing the corrosion resistance of reinforcing steel under aggressive operational conditions using behentrimonium chloride. Scientific Reports.

[71] Wang Y (2015). Effect of pH and chloride on the micro-mechanism of pitting corrosion for high strength pipeline steel in aerated NaCl solutions. Appl. Surf. Sci..

[72] Zeino A, Abdulazeez I, Khaled M, Jawich MW, Obot IB (2018). Mechanistic study of polyaspartic acid (PASP) as eco-friendly corrosion inhibitor on mild steel in 3% NaCl aerated solution. J. Mol. Liq..

[73] El-Taib Heakal F, Fouda AS, Radwan MS (2011). Inhibitive effect of some thiadiazole derivatives on C-steel corrosion in neutral sodium chloride solution. Mater. Chem. Phys..

[74] Abdel Nazeer A, El-Abbasy HM, Fouda AS (2013). Adsorption and Corrosion Inhibition Behavior of Carbon Steel by Cefoperazone as Eco-Friendly Inhibitor in HCl. J. Mater. Eng. Perform..

[75] Hamdy A, El-Gendy NS (2013). Thermodynamic, adsorption and electrochemical studies for corrosion inhibition of carbon steel by henna extract in acid medium. Egypt. J. Pet..

[76] Kannan P, Rao TS, Rajendran N (2018). Improvement in the corrosion resistance of carbon steel in acidic condition using naphthalen-2-ylnaphthalene-2-carboxammide inhibitor. J. Colloid Interface Sci..

[77] Fazayel AS, Khorasani M, Sarabi AA (2018). The effect of functionalized polycarboxylate structures as corrosion inhibitors in a simulated concrete pore solution. Appl. Surf. Sci..

[78] Choi W (2016). Epoxide-functionalization of polyethyleneimine for synthesis of stable carbon dioxide adsorbent in temperature swing adsorption. Nat. Commun..

[79] Xu X, Pejcic B, Heath C, Wood CD (2018). Carbon capture with polyethylenimine hydrogel beads (PEI HBs). J. Mater. Chem. A.

[80] Sharma P, Chakrabarty S, Roy S, Kumar R (2018). Molecular View of CO2 Capture by Polyethylenimine: Role of Structural and Dynamical Heterogeneity. Langmuir.

[81] Zhang W, Liu H, Sun C, Drage TC, Snape CE (2014). Capturing CO2 from ambient air using a polyethyleneimine–silica adsorbent in fluidized beds. Chem. Eng. Sci..

[82] Shen X, Du H, Mullins RH, Kommalapati RR (2017). Polyethylenimine Applications in Carbon Dioxide Capture and Separation: From Theoretical Study to Experimental Work. Energy Technol..

[83] Wang J (2013). Carbon dioxide capture using polyethylenimine-loaded mesoporous carbons. J. Environ. Sci..

[84] Sk MH (2017). Local supersaturation and the growth of protective scales during CO2 corrosion of steel: Effect of pH and solution flow. Corrosion Sci..

[85] Liu QY, Mao LJ, Zhou SW (2014). Effects of chloride content on CO2 corrosion of carbon steel in simulated oil and gas well environments. Corrosion Sci..

[86] Molchan IS (2014). Corrosion behaviour of mild steel in 1-alkyl-3-methylimidazolium tricyanomethanide ionic liquids for CO2 capture applications. RSC Adv..

[87] Emori W, Jiang SL, Duan DL, Zheng YG (2017). Effects of Sodium Thiosulfate and Sodium Sulfide on the Corrosion Behavior of Carbon Steel in an MDEA-Based CO2 Capture Process. J. Mater. Eng. Perform..

